# Porcine cGAS-STING signaling induced autophagy inhibits STING downstream IFN and apoptosis

**DOI:** 10.3389/fimmu.2022.1021384

**Published:** 2022-10-13

**Authors:** Nengwen Xia, Wanglong Zheng, Sen Jiang, Qi Cao, Jia Luo, Jiajia Zhang, Yulin Xu, Shaohua Sun, Kaili Zhang, Nanhua Chen, François Meurens, Jianzhong Zhu

**Affiliations:** ^1^ Comparative Medicine Research Institute, Yangzhou University, Yangzhou, China; ^2^ College of Veterinary Medicine, Yangzhou University, Yangzhou, China; ^3^ Joint International Research Laboratory of Agriculture and Agri-Product Safety, Yangzhou, China; ^4^ Jiangsu Co-innovation Center for Prevention and Control of Important Animal Infectious Diseases and Zoonoses, Yangzhou University, Yangzhou, China; ^5^ Unit of Biology, Epidemiology and Risk Analysis in Animal Health (BIOEPAR), French National Institute for Agriculture, Food, and Environment (INRAE), Oniris, Nantes, France; ^6^ Department of Veterinary Microbiology and Immunology, Western College of Veterinary Medicine, University of Saskatchewan, Saskatoon, SK, Canada

**Keywords:** cGAS-STING pathway, porcine, interferon, autophagy, apoptosis, regulation

## Abstract

The innate immune DNA sensing cGAS-STING signaling pathway has been widely recognized for inducing interferons (IFNs) and subsequent antiviral state. In addition to IFN, the cGAS-STING pathway also elicits other cell autonomous immunity events including autophagy and apoptosis. However, the downstream signaling events of this DNA sensing pathway in livestock have not been well defined. Here, we systematically analyzed the porcine STING (pSTING) induced IFN, autophagy and apoptosis, revealed the distinct dynamics of three STING downstream events, and established the IFN independent inductions of autophagy and apoptosis. Further, we investigated the regulation of autophagy on pSTING induced IFN and apoptosis. Following TBK1-IRF3-IFN activation, STING induced Atg5/Atg16L1 dependent autophagy through LIR motifs. In turn, the autophagy likely promoted the pSTING degradation, inhibited both IFN production and apoptosis, and thus restored the cell homeostasis. Therefore, this study sheds lights on the molecular mechanisms of innate immunity in pigs.

## Introduction

Innate immune response is the first barrier for the host to resist pathogen invasion. It recognizes pathogen associated molecular patterns (PAMPs) or damage associated molecular patterns (DAMPs) through pattern recognition receptors (PRRs), to trigger downstream signaling cascades and exert strong immune responses (1). The innate DNA receptor cGAS recognizes self and non-self double stranded DNA and synthesizes the second messenger 2’3’-cGAMP, which binds STING on endoplasmic reticulum (ER) and triggers the STING translocation from ER. Next, the STING recruits TBK1 which activates IRF3 and NF-κB transcriptions (2, 3). The gene transcriptions generate downstream interferons (IFNs), IFN stimulated genes (ISGs), and proinflammatory cytokines, playing an important antiviral role in virus infections (2, 3). Recently, accumulating evidences have shown that cGAS-STING pathway is involved not only in the IFN induction, but also in autophagy and cell death, which also have anti-virus and/or anti-tumor functions (4, 5).

Autophagy is a primary protective mechanism that allows cells to cope with a series of stressors and to maintain cellular and physiological homeostasis (6). The cGAS-STING pathway has been proved to induce autophagy in mammals, fruit flies and sea anemones, and this function was identified earlier than IFN during evolution (7, 8). Both cGAS and STING induce canonical autophagy as well as non-canonical autophagy (9). STING induces canonical autophagy through ER stress and mammalian target of rapamycin (mTOR) signaling pathway (10). Additionally, STING also induces non-canonical autophagy through the interaction with microtubule associated protein I light chain 3 (LC3) protein (7, 11, 12). STING induced autophagy is usually independent of TBK1 and IRF3 activation, but dependent on Atg5, Atg16L1 and Atg7 (11–13).

STING has been reported to be involved in regulating several types of cell death, including apoptosis, necroptosis, pyroptosis, ferroptosis and so on (4, 14). Apoptosis, also known as the non-inflammatory type of programmed cell death, is one of the most widely studied cell death pathways (15). At present, the mechanism of STING induced apoptosis is not clear; nevertheless, most studies point to ER stress (16–20). Under different conditions including agonist stimulation, gain-of-function mutation, bacterial infection or ethanol treatment, STING and ER stress are linked to trigger apoptosis, through an evolutionarily conserved unfolded protein response (UPR) motif (18). Alternatively, STING may induce apoptosis through downstream IRF3 or NF-κB (16, 17, 21, 22), where STING mainly mediates the phosphorylation of IRF3 and triggers the formation of IRF3-Bax complex (16, 17, 21).

Although the cGAS-STING induced autophagy and apoptosis in humans and mice have been extensively studied, the mechanisms in an important animal species such as the pig are still unknown or unclear. Further, as the STING downstream signaling events, IFN, autophagy and apoptosis all play a role in anti-virus or anti-tumor immunity, it is thus necessary to clarify the relationships between all these events induced by STING. In this study, we found that porcine STING (pSTING) signaling activates IFN, autophagy and apoptosis with the peak levels appearing in sequence, with autophagy and apoptosis inductions independently of IFN. In addition, autophagy inhibited both IFN production and apoptosis occurrence, restoring the cell homeostasis.

## Materials and methods

### Chemical reagents and antibodies

TRIpure Reagent for RNA extraction was obtained from Aidlab (Beijing, China). 2×Taq Master Mix (Dye plus) were from Vazyme Biotech Co.,Ltd (Nanjing, China). Poly I:C-LMW, 2’3’-cGAMP and poly dA:dT were bought from InvivoGen (Hong Kong, China). The Golden Star T6 Super PCR mix polymerase was from Tsingke (Nanjing, China) and the KOD plus neo polymerase was from Toyobo (Shanghai, China). Annexin V/propidium iodide (PI) were purchased from Becton Dickinson Company (BD; Franklin Lakes, NJ, USA).

The rabbit mAbs of HA (3724S), LC3I/II (3868), cleaved caspase3 (Asp175) (9664S), TBK1 (3504S), p-TBK1 (5483S), IRF3 (11904S), FLAG (14793), GFP (2956) and β-actin (5057) were acquired from Cell Signaling Technology (Boston, MA, USA). The phosphorylated-IRF3 (Ser396) rabbit mAb (MA5-14947), Goat anti-rabbit IgG (H+L) cross-adsorbed 488 (35553) were from ThermoFisher Scientific (Shanghai, China), The mCherry rabbit pAb (ab183628) and Goat anti-mouse IgG H&L Alexa Fluor^®^594 (ab150120) were acquired from Abcam (Cambridge, UK). The STING rabbit pAb (19851-1-AP), Bax rabbit pAb (50599-2-lg), Bcl2 mouse mAb (60178-1-lg), Atg5 rabbit pAb (10181-2-AP), Atg16L1 mouse mAb (67943-1-Ig), were all purchased from ProteinTech (Wuhan, China). The HA mouse mAb, GFP mouse mAb, HRP anti-mouse IgG (HS201), and HRP anti-rabbit IgG (HS101) were all acquired from Transgen Biotech (Beijing, China).

### Cells and cell transfection

HEK293T was cultured in DMEM (Hyclone Laboratories, USA) containing 10% fetal bovine serum (FBS) and 100 IU/mL of penicillin plus 100 mg/ml streptomycin. Porcine alveolar macrophages (3D4/21, ATCC CRL-2843™) were cultured in RPMI (Hyclone Laboratories) containing 10% FBS with penicillin/streptomycin. All cells were maintained at 37°C with 5% CO2 in a humidified incubator. Cell transfection was performed by using the Lipofectamine 2000 (ThermoFisher Scientific, Shanghai, China) following the manufacturer’s instructions. The Vesicular Stomatitis Virus (VSV-GFP) and Herpes Simplex Virus-1 (HSV-1-GFP) were both provided by Dr. Tony Wang in SRI International USA, and used as we described previously (23).

### STING molecular cloning and gene mutations

The HA tagged pcDNA DEST^47^ plasmids of pcGAS and pSTING were previously constructed and have been used in our laboratory (24). The pSTING was PCR amplified from pcDNA DEST^47^-pSTING-2HA using 2×Taq Master Mix (Dye plus) and the designed primers as presented in **Supplementary Table S1**. The PCR product was cloned into the *Bgl* II and *EcoR* I sites of pmCherry-C1 vector. The mutation PCR primers of pSTING were designed by QuickChange Primer Design method (https://www.agilent.com) and shown in **Supplementary Table S1**. The mutation PCR was performed with KOD plus neo polymerase and pcDNA-DEST^47^-pSTING-2HA or pmCherry-C1-pSTING as the templates. The PCR products were transformed into competent DMT *E.coli* after *Dpn* I digestion, and the resultant mutants were sequence confirmed.

### Preparation of homozygous KO 3D4/21 cell clones by CRISPR-Cas9 approach

The CRISPR gRNAs targeting porcine Atg5 and Atg16L1 were designed using the web tool from Benchling (www.benchling.com). For each gene, two pairs of gRNAs were selected according to the prediction score. The DNA sequences encoding all gRNA are shown in **Supplementary Table S2**. The annealed gRNA encoding DNA sequences were cloned into the *Bbs*I site of pX458-EGFP and the recombinant pX458-gRNA plasmids were sequence confirmed. 3D4/21 cells grown in 6-well plates (6-8×10^5^ cells/well) were co-transfected with each pX458-gRNA using Lipofectamine 2000. Twenty four hours later, the GFP positive cells were sorted by flow cytometry and grown in 96-well plates by limiting dilution for monoclonal growth. The single clones of 3D4/21 cells were used to detect the protein expression of Atg5 or Atg16L1 by Western blotting. All 3D4/21 cell clones were detected for genomic DNA editing by PCR using primers shown in **Supplementary Table S2**. Specifically, the genomic PCR products were cloned into T vector using pClone007 versatile simple vector kit (TsingKe Biological Technology, Beijing, China) and inserted fragments were sequenced and analyzed for base insertion and deletion (ins/del) mutations. Finally, homologous KO cell clones were obtained for both Atg5 and Atg16L1, respectively.

### RNA extraction and reverse transcription-quantitative PCR

Total RNA was extracted using TRIpure reagent following the manufacturer’s suggestions. The extracted RNA was reverse transcribed into cDNA using HiScript 1^st^ strand cDNA synthesis kit, and then the target gene expressions were measured by quantitative PCR with SYBR qPCR master Mix (Vazyme, Nanjing, China) in StepOne Plus equipment (Applied Biosystems). The qPCR program is denaturation at 94°C for 30 s followed by 40 cycles of 94°C for 5 s and 60°C for 30 s. The relative mRNA levels were normalized to β-actin mRNA levels, and calculated using 2^−ΔΔCT^ method. The sequence of qPCR primers used are shown in **Supplementary Table S3**.

### Western blotting detection

Whole cell proteins were extracted with an RIPA lysis buffer. Then, the concentration of lysate protein was analyzed and adjusted using the BCA protein assay kit (Beyotime Institute of Biotechnology, Shanghai, China). The protein samples were mixed at the ratio of 3:1 with 4 × loading buffer and boiled for 10 min. The protein supernatants were run by SDS-PAGE, and then the proteins in the gel were transferred to PVDF membranes. The membranes were incubated with 5% skim milk solution at room temperature (RT) for 2 h, probed with the indicated primary antibodies at 4°C overnight, washed, and then incubated with secondary antibodies for 1 h at RT. The protein signals were detected by ECL detection substrate and imaging system.

### Cell apoptosis analysis

The level of cell apoptosis was examined using the Annexin V-fluorescein isothiocyanate (FITC) apoptosis detection kit. Briefly, after treatment, the cells were harvested and washed with the binding buffer, and then resuspended in the binding buffer. The staining solutions of Annexin V-FITC and PI were added one by one. The cells were incubated at RT for 15 min in the dark, and the stained cells immediately detected using flow cytometry. The ratios of Annexin positive cells including PI negative and positive were calculated as the levels of apoptotic cells.

### Confocal microscopy

3D4/21 cells grown on 15 mm glass bottom cell culture dish (NEST, Wuxi, China) were transfected with pSTING-HA or pSTING mutants and pCMV-GFP-LC3II (D2815, Beyotime, Shanghai, China), using Lipofectamine 2000. Twenty-four hours later, the cells were fixed with 4% paraformaldehyde at RT for 30 min, and permeabilized with 0.5% Triton X-100 for 20 min. After washing with PBS, the cells were sequentially incubated with primary LC3I/II rabbit pAb (1:200) and HA mouse mAb (1:200), and next secondary Donkey anti-rabbit IgG Alexa Fluor 488 (1:800), Goat anti-mouse IgG H&L Alexa Fluor 594 (1:800). The stained cells were counter-stained for cell nucleus with 0.5mg/mL 4’,6-diamidino-2-phenylindole (DAPI, Beyotime, China) at 37°C for 15 min. Lastly, cells were visualized under laser-scanning confocal microscope (LSCM, Leica SP8, Solms, Germany) at the excitation wavelengths 488 nm and 594 nm, respectively.

### Statistical analysis

All of the experiments were representative of two or three similar experiments. The results were analyzed by using SPSS and presented as the mean ± standard deviation (SD) of triplicate samples (n=3). Statistical analysis was performed by using Student’s *t*-test and *ANOVA*. * denotes *p* < 0.05, ** denotes *p* < 0.01 and *p* < 0.05 is considered statistically significant.

## Results

### Activation of porcine cGAS-STING signaling pathway induces autophagy

**Figure 1:**
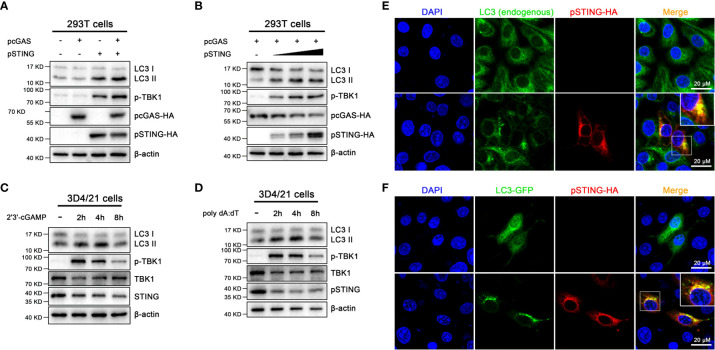
Activation of porcine cGAS-STING signal causes autophagy. **(A)** 293T cells were transfected with the indicated pcDNA encoded pcGAS (0.5 μg), pSTING (0.5 μg) with HA tags. **(B)** 293T cells were co-transfected with pcGAS (0.5 μg) and pSTING at different amounts (0, 0.125, 0.25 and 0.5 μg) for 24 h **(C, D)** 3D4/21 cells were stimulated by transfection of 2’3’-cGAMP or poly dA:dT (1 μg/mL) for the indicated times. Western blotting were performed to detect the LC3I/II protein expressions. **(E)** 3D4/21 cells were transfected with the pcDNA-pSTING-2HA (1 μg) for 24 h **(F)** 3D4/21 cells were co-transfected with pcDNA-pSTING-2HA and LC3-GFP for 24 h The confocal microscope was used to analyze the formations of endogenous and exogenous LC3 puncta, indicators of phagosomes. The small insets are magnified on the up-right corner. Scale bar: 20 μm.

First, we expressed porcine cGAS (pcGAS) and porcine STING (pSTING) in transfected 293T cells and the various cell signaling pathways were examined by Western blotting (**Figure 1A** and **Figure S1**). The expression of pSTING but not pcGAS activated the phosphorylation of TBK1 (p-TBK1), indicating an IFN response, and the co-expression of pcGAS intensified the pSTING activated p-TBK1 (**Figure 1A**). Similarly, the ectopic pSTING expression induced autophagy as evidenced by the conversion of LC3I to LC3II, the indicator of LC3 lipidation and the co-expression of pcGAS further enhanced the LC3 lipidation (**Figure 1A**). In addition, pSTING activated both p-TBK1 and LC3 lipidation in a pSTING dose dependent manner (**Figure 1B** and **Figure S1**).

Second, porcine macrophages 3D4/21 were stimulated by the transfection of STING specific agonist 2’3’-cGAMP (**Figure 1C** and **Figure S1**) or cGAS agonist poly dA:dT (**Figure 1D** and **Figure S1**), and the p-TBK1 and LC3 lipidation were examined. The 2’3’-cGAMP activated p-TBK1 with peaking level at 2 h post stimulation, whereas the LC3 lipidation peaked at 4 h post stimulation (**Figure 1C**). Similarly, poly dA:dT activated p-TBK1 and LC3 lipidation with peak levels at 2 and 4 h post stimulation, respectively (**Figure 1D**).

Third, the autophagy induction was further validated by observation for LC3 autophagosome formations under fluorescence microscopy (**Figures 1E, F**). In normal 3D3/21 cells, both endogenous and exogenous LC3 were diffusely distributed in cytoplasm; upon ectopic pSTING expression, both endogenous and exogenous LC3 presented as puncta indicative of autophagosome formations, which were also co-localized with ectopic pSTING (**Figures 1E, F**).

### Activation of porcine cGAS-STING signaling pathway induces apoptosis

**Figure 2:**
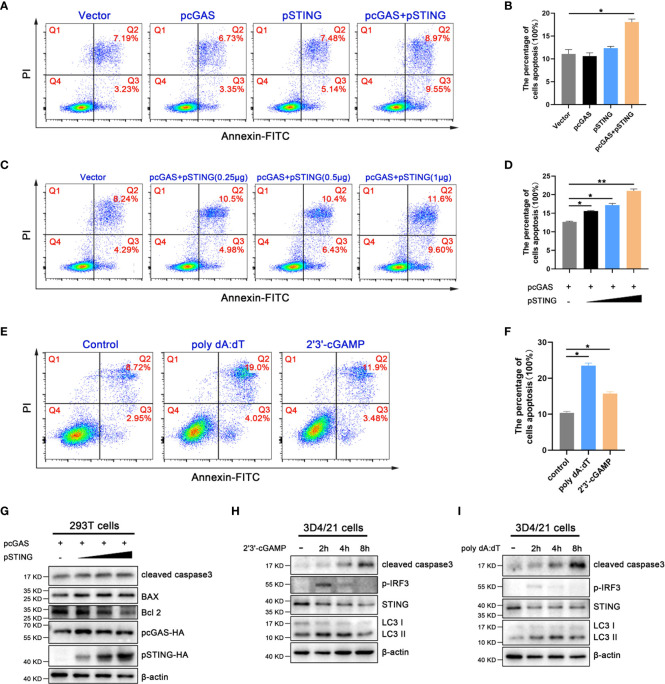
Activation of porcine cGAS-STING signal induces apoptosis. **(A, B)** 293T cells were transfected with pcDNA encoded pcGAS (0.5 μg), pSTING (0.5 μg) as indicated. **(C, D)** 293T cells were co-transfected with pcGAS (0.5 μg) plus pSTING at different amounts (0, 0.125, 0.25 and 0.5 μg). **(E, F)** 3D4/21 cells were stimulated by transfection of 2’3’-cGAMP (1 μg/mL) or poly dA:dT (1 μg/mL) for 16 h. Cells were stained with Annexin V and PI followed by flow cytometry analysis. The percentages of cells in each quadrants of dot blots were indicated and apoptotic cell levels were presented in graphs. *p < 0.05 and **p < 0.01. **(G)** 293T cells were co-transfected with pcGAS (0.5 μg) plus pSTING at different amounts (0, 0.125, 0.25 and 0.5 μg) for 24 h. **(H, I)** 3D4/21 cells were stimulated with 2’3’-cGAMP or poly dA:dT (1 μg/mL) for the indicated times. Cells were analyzed by Western blotting for cleaved caspase 3 expressions.

We found that the activation of porcine cGAS-STING signaling pathway seriously affected cell growth; therefore, the effects of cGAS-STING signaling on cell apoptosis in 293T and 3D4/21 cells were analyzed using annexin V staining followed by flow cytometry. As shown in **Figures 2A, B**, the ratios of cell apoptosis in pcGAS and pSTING co-transfected 293T cells was significantly higher relative to control transfected cells. Further, the apoptosis levels increased in the pSTING concentration dependent manner (**Figures 2C, D**). In 3D4/21 cells, both 2’3’-cGAMP and poly dA:dT stimulations triggered significantly higher levels of apoptosis, with poly dA:dT stimulating more potent apoptosis (**Figures 2E, F**).

Bcl2 family plays a key role in apoptosis (25), and it is composed of pro-apoptotic members such as Bax and anti-apoptotic members such as Bcl2. The Bax and Bcl2 regulate the mitochondrial outer membrane permeabilization (MOMP), which is the key to induce intrinsic apoptosis (26). Caspase 3, one of the important members of caspase family, is the executor of apoptosis (26). Western blotting showed that the cleaved caspase 3 and the proportion of Bax/Bcl2 markedly increased in a pSTING dose dependent manner in pcGAS/pSTING co-transfected 293T cells (**Figure 2G** and **Figure S2**). In 3D4/21 cells, both 2’3’-cGAMP and poly dA:dT stimulations activated the cleaved caspase 3, with the peak levels at 8 h post stimulation, following those of p-IRF3 indicative of IFN induction (2 h post stimulation) and LC3 lipidation indicative of autophagy (4 h post stimulation) (**Figures 2H, I** and **Figure S2**).

### Porcine STING is essential for autophagy and apoptosis inductions

**Figure 3:**
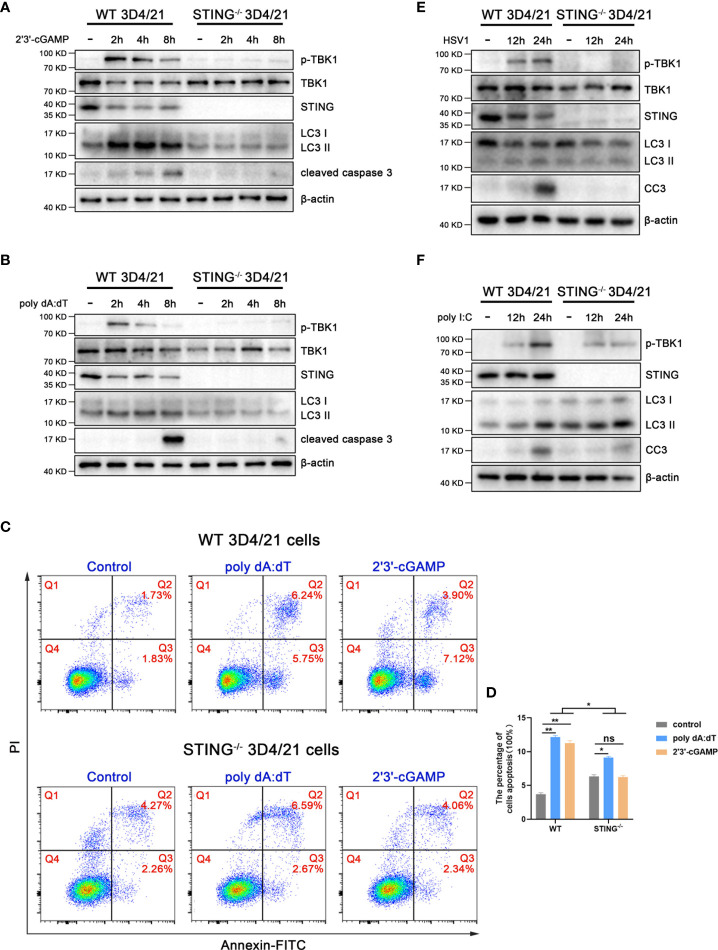
STING is essential for autophagy and apoptosis. STING^-/-^ and wild type 3D4/21 cells were stimulated by 2’3’-cGAMP (1 μg/mL) **(A)**, poly dA:dT (1 μg/mL) **(B)**, HSV1 (MOI=0.01) **(E)** or poly I:C (1 μg/mL) **(F)**, respectively. Cells were harvested at the indicated times and analyzed by Western blotting. The abbreviations CC3 in **(E)**, **(F)** and subsequent Figures all denote cleaved caspase 3. **(C, D)** Cells were stimulated with 2’3’-cGAMP or poly dA:dT for 16 h, and the stimulated cells were stained with annexin V and PI followed by flow cytometry for cell apoptosis analysis. ns, not significant; *p < 0.05 and **p < 0.01.

To check the role of pSTING in the inductions of IFN, autophagy and apoptosis, the STING^-/-^ 3D4/21 cells were used and stimulated with various agonists including 2’3’-cGAMP for STING, poly dA:dT and HSV1 for cGAS, and poly I:C for RIG-I/MDA5, and the downstream cell events were examined by Western blotting (**Figure 3** and **Figure S3**). Consistently, in normal 3D4/21 cells, the IFN response reflected by p-TBK1 appeared and peaked at 2 h post stimulations with 2’3’-cGAMP (**Figure 3A**) or poly dA:dT (**Figure 3B**); the autophagy induction evidenced by LC3 lipidation peaked at 4 h post stimulations with 2’3’-cGAMP (**Figure 3A**) or poly dA:dT (**Figure 3B**); the apoptosis induction indicated by cleaved caspase 3 peaked at 8 h post stimulations with 2’3’-cGAMP (**Figure 3A**) or poly dA:dT (**Figure 3B**). Whereas in STING^-/-^ 3D4/21 cells, the p-TBK1, LC3 lipidation and cleaved caspase 3 all disappeared with both 2’3’-cGAMP and poly dA:dT stimulations (**Figures 3A, B**). The results of flow cytometry further showed that 2’3’-cGAMP and poly dA:dT induced apoptosis in STING^-/-^ 3D4/21 cells were largely reduced relative to normal 3D4/21 cells (**Figures 3C, D**).

HSV1 activated cGAS-STING to induce p-TBK1, LC3 lipidation and cleaved caspase 3 in normal 3D4/21 cells, and the three cell events all disappeared in STING^-/-^ 3D4/21 cells (**Figure 3E**). However, the poly I:C, as control agonist to activate RNA sensors RIG-I and/or MDA5, induced p-TBK1, LC3 lipidation and cleaved caspase 3 in both normal 3D4/21 cells and STING^-/-^ 3D4/21 cells (**Figure 3F**). Taken together, these results suggest that porcine cGAS-STING signaling pathway induces IFN, autophagy and apoptosis with different dynamics, and pSTING is essential for the inductions of three cellular events.

### The pSTING induced apoptosis and autophagy are both IFN independent

**Figure 4:**
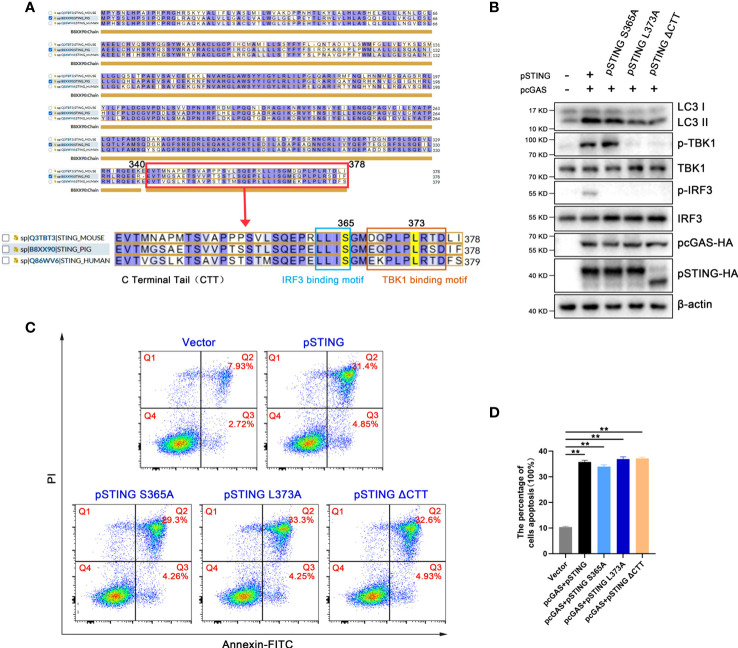
The apoptosis and autophagy induced by STING signaling are both IFN independent. **(A)** The alignment of human, mouse and porcine STING with CTT domain enlarged, IRF and TBK1 recruitment motifs box marked. The pSTING S365 in IRF recruitment motif and L373 in TBK1 recruitment motif are also indicated. **(B, D)** 293T cells were co-transfected with pcDNA-pcGAS-2HA (0.5 μg) plus pcDNA-pSTING-2HA, pSTING S365A, pSTING L373A or pSTING ΔCTT (each 0.5 μg) for 24 h, followed by Western blotting **(B)** and flow cytometry **(C, D)**. **p < 0.01.

It has been known that STING is able to induced IFN independent autophagy (7), and previous studies showed that STING regulates apoptosis through downstream IRF3, but with dispute on the involvement of IFN (16, 27, 28). It has been reported that human STING serine 366 and leucine 374 (equivalent to pSTING S365 and L373) are critical sites for its recruitment and activation of IRF3 and TBK1, respectively, and thus important for subsequent IFN induction (29, 30). The C-terminal tail (CTT) of STING (pSTING residues 340-378), contains both IRF3 and TBK1 recruitment and activation motifs (**Figure 4A**), are critical for IFN induction (31).

In order to explore whether the inductions of autophagy and apoptosis by STING requires the participation of IFN, we constructed pSTING mutants STING S365A, L373A and ΔCTT, and examined the cellular events in transfected 293T cells by Western blotting (**Figure 4B** and **Figure S4**). Consistent with the results in humans and mice, pSTING S365A could not phosphorylate IRF3, but still phosphorylated TBK1, whereas L373A and ΔCTT could phosphorylate neither one (**Figure 4B**). On the contrary, all three IFN defective pSTING mutants was able to induce autophagy indicated by LC3 lipidation (**Figure 4B**). In the meantime, these IFN defective pSTING mutants induced similar levels of apoptosis to wild type pSTING as shown by flow cytometry (**Figures 4C, D**). Together, these results suggest that pSTING induced autophagy and apoptosis are both IFN independent.

### The pSTING exerts IFN independent antiviral effect, likely *via* autophagy and/or apoptosis

**Figure 5:**
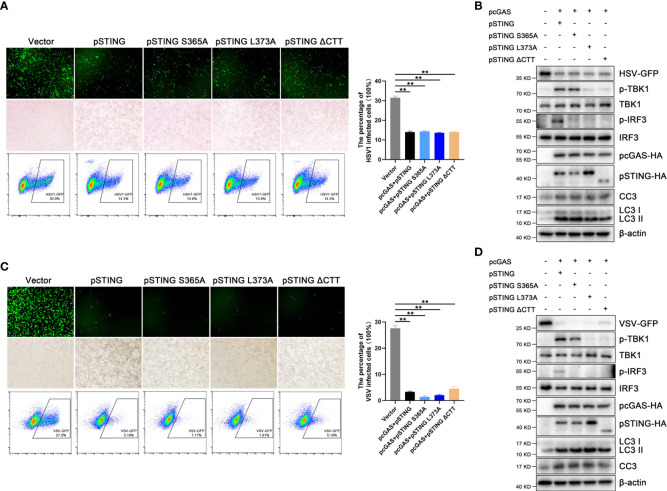
STING exerts antiviral effects independent of IFN. **(A, B)** 293T cells were co-transfected with pcDNA-pcGAS-2HA (0.5 μg) plus pcDNA-pSTING-2HA or various mutants (each 0.5 μg) for 24 h, followed by HSV1 (MOI=0.01) infection for another 12 h. **p < 0.01. **(C, D)** 293T cells were co-transfected with pcDNA-pcGAS-2HA (0.5 μg) and pcDNA-pSTING-2HA or various mutants (each 0.5 μg) for 24 h, followed by VSV (MOI=0.001) infection for another 8 h. Cells were analyzed for viral GFP signal by fluorescence microscopy, viral GFP intensity by flow cytometry and protein expressions by Western blotting. **p < 0.01.

It was reported that STING induced autophagy mediate antiviral function (8), and we were curious to know whether the IFN defective pSTING mutants exert antiviral function. In 293T cells, the pSTING mutants S365A, L373A and ΔCTT were co-transfected with pcGAS into 293T cells and subsequently infected by HSV1 or VSV. Surprisingly, all the IFN defective pSTING mutants exhibited similar antiviral effects against HSV1 (**Figures 5A, B**) and VSV (**Figures 5C, D**) as wild type pSTING, which was evidenced by viral GFP fluorescence microscopy (**Figures 5A, C**), viral GFP intensity in flow cytometry (**Figures 5A, C**), and GFP immunoblotting (**Figures 5B, D** and **Figure S5**). The IFN defective pSTING mutants did not induce p-TBK and/or p-IRF3, again confirming the absence of IFN response, whereas these pSTING mutants exhibited normal inductions of LC3 lipidation and cleaved caspase 3 (**Figures 5B, D**). These results suggest that pSTING exerts its antiviral function likely *via* autophagy and/or apoptosis.

### The pSTING induced autophagy suppresses its IFN response

**Figure 6:**
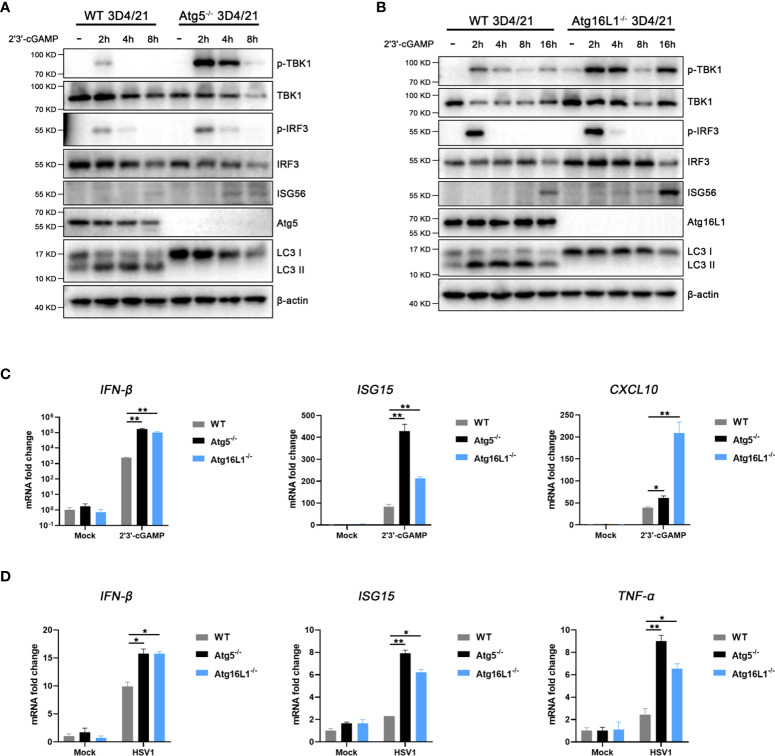
STING induced autophagy suppresses IFN signaling. **(A, B)** Wild type and Atg5^-/-^ 3D4/21 cells **(A)** or Atg16L1^-/-^ 3D4/21 cells **(B)** were stimulated by transfection of 2’3’-cGAMP (1 μg/mL) for the indicated times, and cell lysates were analyzed by Western blotting with the indicated antibodies. **(C, D)** Wild-type, Atg5^-/-^ and Atg16L1^-/-^ 3D4/21 cells were stimulated with 2’3’-cGAMP (1 μg/mL) for 4h **(C)** or infected with HSV1 (0.01 MOI) for 8 h **(D)**. The harvested cells were analyzed by RT-qPCR for downstream gene expressions as indicated. *p < 0.05 and **p < 0.01.

In order to study the influence of pSTING induced autophagy on IFN response, we constructed two autophagy related gene knockout cells, Atg5^-/-^ and Atg16L1^-/-^ 3D4/21 cells, respectively (**Figure S9**). Previous studies have shown that STING induced autophagy is Atg5 and Atg16L1 dependent (7, 11, 12), and our results showed that 2’3’-cGAMP stimulated LC3 lipidation disappeared in both Atg5^-/-^ 3D3/21 cells (**Figure 6A** and **Figure S6**) and Atg16L1^-/-^ 3D4/21 cells (**Figure 6B** and **Figure S6**). In contrast, the levels of p-TBK, p-IRF3 and downstream ISG56 were all upregulated in both gene knockout macrophages (**Figures 6A, B**), suggesting the negative regulation of IFN response by autophagy.

Further, the mRNA transcriptions of IFNβ and co-regulated genes were examined by RT-qPCR. Upon 2’3’-cGAMP stimulation, the activated IFNβ, ISG15 and CXCL10 mRNA levels were significantly upregulated in both Atg5^-/-^ 3D3/21 cells and Atg16L1^-/-^ 3D4/21 cells relative to those in wild type 3D4/21 cells (**Figure 6C**). Upon HSV1 infection, the activated IFNβ, ISG15 and TNFα mRNA levels were also significantly upregulated in both Atg5^-/-^ 3D3/21 cells and Atg16L1^-/-^ 3D4/21 cells relative to those in wild type 3D4/21 cells (**Figure 6D**). These results suggest that pSTING induced autophagy plays a negative regulatory role in its IFN response.

### The pSTING induced autophagy inhibits its apoptosis activity

**Figure 7:**
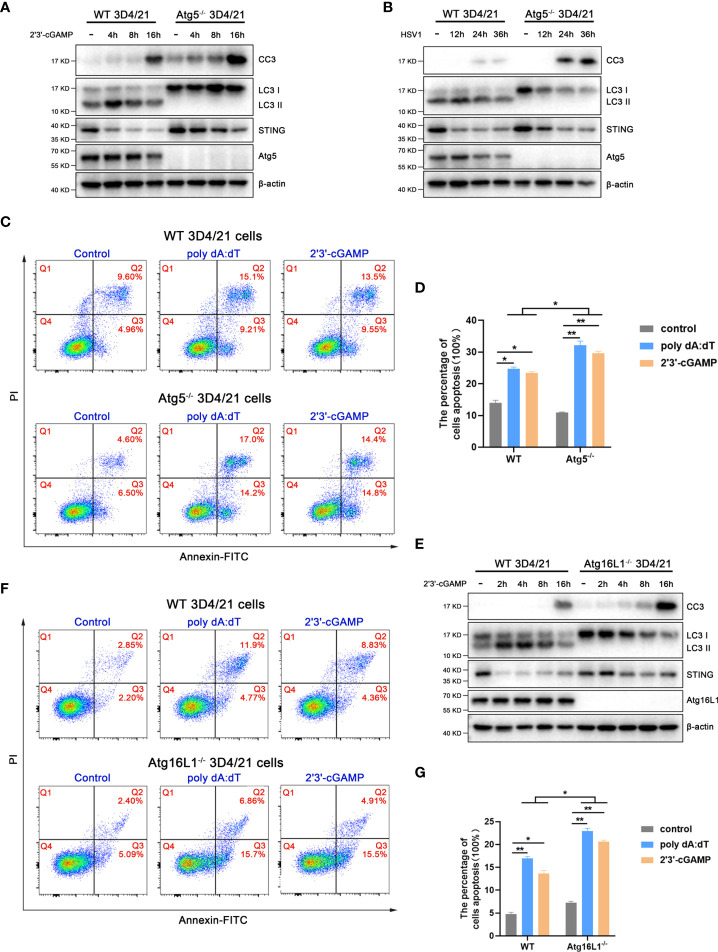
STING induced autophagy promotes STING degradation and inhibits apoptosis. **(A, B)** Wild type and Atg5^-/-^ 3D4/21 cells were stimulated by 2’3’-cGAMP (1 μg/mL) **(A)** or HSV1 (0.01 MOI) **(B)** for the indicated times, followed by Western blotting. **(C, D)** Wild type and Atg5^-/-^ 3D4/21 cells were stimulated by poly dAdT or 2’3’-cGAMP (each 1 μg/mL) for 16 h, followed by flow cytometry analysis of cell apoptosis. *p < 0.05 and **p < 0.01. **(E)** Wild type and Atg16L1^-/-^ 3D4/21 cells were stimulated by 2’3’-cGAMP (1 μg/mL) for the indicated times, followed by Western blotting. **(F, G)** Wild type and Atg16L1^-/-^ 3D4/21 cells were stimulated by poly dA:dT or 2’3’-cGAMP (each 1 μg/mL) for 16 h, followed by flow cytometry analysis of cell apoptosis. *p < 0.05 and **p < 0.01.

In order to analyze the effect of pSTING activated autophagy on apoptosis, we first examined the caspase 3 cleavage in Atg5^-/-^ 3D4/21 cells by Western blotting (**Figures 7A, B** and **Figure S7**). The levels of cleaved caspase 3 were greatly upregulated in Atg5^-/-^ 3D4/21 cells upon 2’3’-cGAMP stimulation (**Figure 7A**) or HSV1 infection (**Figure 7B**). Similarly, both poly dA:dT and 2’3’-cGAMP induced cell apoptosis were significantly upregulated in Atg5^-/-^ 3D3/21 cells analyzed by flow cytometry (**Figures 7C, D**).

Simultaneously, the levels of cleaved caspase 3 were also obviously upregulated in Atg16L1^-/-^ 3D3/21 cells upon 2’3’-cGAMP stimulation (**Figure 7E** and **Figure S7**). Further, both poly dA:dT and 2’3’-cGAMP induced cell apoptosis were significantly upregulated in Atg16L1^-/-^ 3D4/21 cells as observed by flow cytometry (**Figures 7F, G**). These results suggest that pSTING induced autophagy plays a negative regulatory role in its apoptosis activity.

### LIR motifs participate in pSTING mediated autophagy induction and regulation

**Figure 8:**
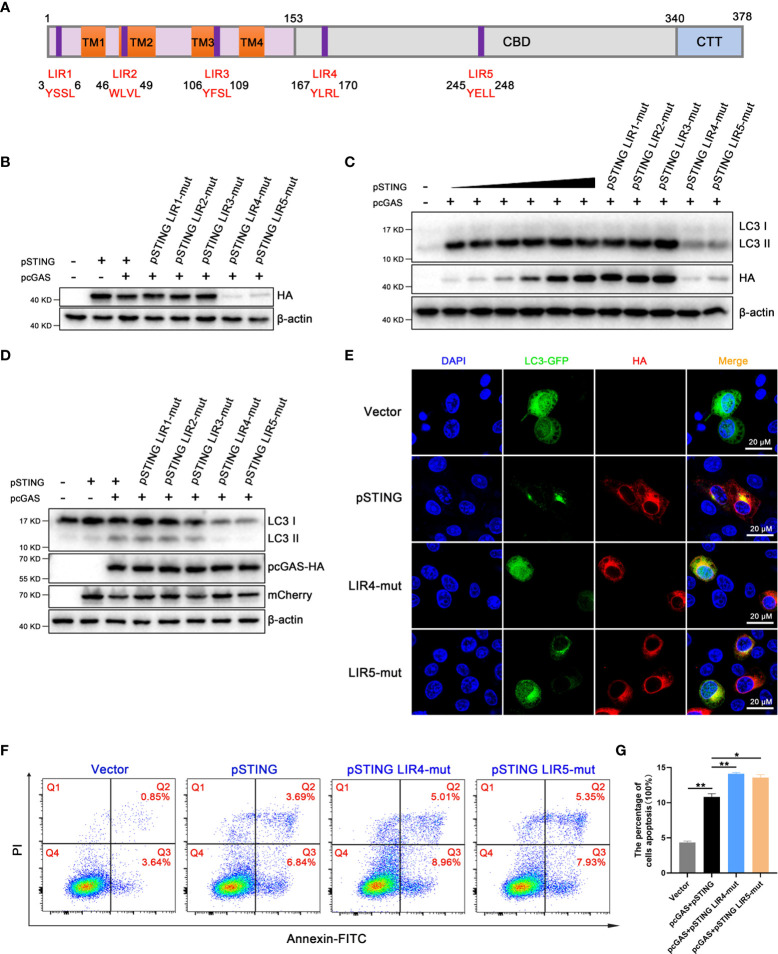
LIRs as important motifs participate in STING mediated autophagy regulation. **(A)** Graphical representation of the pSTING showing the five potential LIR motifs. The N-terminal domain contains 4 transmembrane regions (TM1-4), the CBD is c-di-GMP binding domain, and the C-terminal tail (CTT) contains the cytoplasmic tail. The amino acid sequences of the predicted LIR motifs are indicated. **(B)** 293T cells were transfected with pcDNA-pSTING-2HA or corresponding LIR mutants (1μg each) for 24 h, followed by Western blotting. **(C)** 293T cells were co-transfected with pcDNA-pcGAS-2HA (0.5 μg) and pcDNA-pSTING-2HA (0, 0.05, 0.1, 0.2, 0.3, 0.4, 0.5 μg) or corresponding LIR mutants (each 0.5 μg) for 24 h, followed by Western blotting. **(D)** 293T cells were co-transfected with pcDNA-pcGAS-2HA (0.5 μg) plus pmCherry-C1-pSTING or corresponding LIR mutants (0.5 μg) for 24 h, followed by Western blotting. **(E)** 3D4/21 cells were co-transfected with LC3-GFP (0.5 μg) and pSTING-2HA or LIR4-mut or LIR5-mut (0.5 μg each) for 24 h. Images of LC3 and pSTING were then captured by confocal microscopy. Scale bar: 20 μm. **(F, G)** 293T cells were co-transfected with pcGAS-HA (0.5 μg) plus pSTING-HA (0.05 μg), LIR4-mut or LIR5-mut (0.5 μg) for 24 h, and the Annexin V positive cells were detected by flow cytometry. *p < 0.05 and **p < 0.01.

A recent study identified STING as an autophagy receptor, which interacts with LC3 through LIR motifs (11). LIR, called LC3 interaction region, is a typical linear amino acid motif, with conserved sequence W/YxxL/I (32). Using the iLIR server to predict potential LIR motifs in the porcine STING protein (33), we identified five hypothetical LIR motifs, with LIRs 1-3 in the transmembrane region of pSTING and LIR4 and 5 in the cyclic dinucleotide-binding domain (CBD) (**Figure 8A**). To verify the roles of LIR motifs in pSTING induced autophagy, we constructed five LIR motif mutants of pSTING, with the first residue W/Y and the fourth residue L/I in each LIR motif mutated into A, and verified the protein expressions. Western blotting showed that the pcDNA recombinant pSTING LIR4 and LIR5 mutants had very poor protein expressions in transfected 293T cells (**Figure 8B** and **Figure S8**). We expressed the pSTING with different doses and ensured the comparable protein expressions of pSTING to various LIR mutants (**Figure 8C** and **Figure S8**). Based on comparable protein expression levels, it was concluded that pSTING LIR4 and LIR5 motifs are important for pSTING induced autophagy as indicated by LC3 lipidation (**Figure 8C** and **Figure S8**). The pmCherry recombinant pSTING LIR mutants further supported the hypothesis that LIR4 and LIR5 motifs are important for pSTING induced autophagy (**Figure 8D** and **Figure S8**). Accordingly, the laser confocal microscopy results showed that the accumulations of LC3 puncta in 3D4/21 cells were greatly reduced in LIR4 or LIR5 mutant relative to wild type pSTING. (**Figure 8E**). Importantly, we found that both LIR4 and LIR5 mutants induced significantly heightened apoptosis relative to wild type pSTING in transfected 293T cells when the pSTING transfection amount was reduced to ensure the equal protein expression as LIR4 and LIR5 mutants (**Figures 8F, G**), which is consistent with the results from Agt5^-/-^ and Atg16L1^-/-^ macrophages (**Figure 7**), suggesting that pSTING induced autophagy negatively regulates apoptosis.

## Discussion

In this study, we first found that porcine cGAS-STING signaling activation not only induces TBK1-IRF3 mediated IFN response, but also activates Atg5/Atg16L1 dependent autophagy and caspase3 cleavage mediated apoptosis. The IFN, autophagy and apoptosis downstream of pSTING peak in a sequential order. Second, both autophagy and apoptosis inductions are IFN independent, suggesting no regulation of IFN signaling on autophagy and apoptosis. Third, pSTING activated Atg5/Atg16L1 dependent autophagy is LIR motif related and exerts a negative regulation on IFN and apoptosis. Thus, we define the pSTING downstream IFN, autophagy and apoptosis, and are able to make a conclusion for the interrelationship of pSTING downstream IFN, autophagy and apoptosis, i.e. IFN does not regulate autophagy and apoptosis, and autophagy can negatively regulate IFN and apoptosis.

It is known that STING induces autophagy independently of TBK1-IRF3 activation and IFN signaling (7, 10–12, 34). We confirmed the IFN independent induction of autophagy by pSTING (**Figure 4**). There are 7 LIR motifs in human STING and LIR4, LIR6 and LIR7 are important for STING autophagy (11). Although only 5 predicted LIR motifs exist in pSTING with LIR1 unique to pSTING, we found that LIR4 and LIR5 in pSTING are associated with autophagy (**Figure 8**) which correspond to LIR 4 and LIR7 in hSTING. The coincident results reflect the conserved function between pigs and human STING.

Whether STING induces apoptosis independently of TBK1-IRF3 activation and IFN signaling is unknown (16–21). Some studies suggested that ER stress is related with STING induced apoptosis (16–20), whereas others showed that the activation of TBK1-IRF3 is required (16, 17, 21). Our results using several IFN defective pSTING mutants including S365A, L373A and ΔCTT clearly showed that the apoptosis induced by pSTING is also independent of TBK1-IRF3 phosphorylation and activation (**Figure 4**). However, the pSTING induced apoptosis in TBK1^-/-^ and IRF3^-/-^ 3D4/21 cells are largely decreased, suggesting the presence of TBK1 and IRF3 is necessary for the apoptosis (data not shown). The exact mechanism of pSTING induced apoptosis deserves further investigation. Since pSTING induced autophagy and apoptosis are both IFN independent (**Figure 4**), autophagy and/or apoptosis likely mediate the IFN independent antiviral function of pSTING (**Figure 5**). Our separate study further showed pSTING exerts an antiviral function independently of both IFN and autophagy, therefore, the antiviral activity of STING appears complex.

Autophagy is originally a self-protection measure for cells to cope with harsh environments such as hunger, stress or pathogen infections (35, 36). Our data showed that it plays a negative regulatory role in the pSTING induced activations of TBK1 and IRF3, and IFN response at early stage of STING activation, as well as in the pSTING induced apoptosis at the late stage of STING activation, thus maintaining cell homeostasis after STING activation. STING has multiple negative feedback regulation mechanisms after activation, including ubiquitin proteasome pathway (37, 38), lysosomal degradation pathway (39) and autophagy mediated degradation (40, 41). Here we observed the pSTING degradation post stimulations, which is accompanied by autophagy (**Figures 1** - **3** and **Figure 7**). Further, under the autophagy knockout conditions, the pSTING degradation weakened, which might be responsible for the heightened IFN response and apoptosis (**Figure 6** and **7**). Therefore, autophagy may exert homeostatic role by promoting pSTING degradation.

In summary, our work suggests that STING triggered IFN independent activity including autophagy and apoptosis have significant regulatory roles in STING signaling activity. Autophagy plays a negative regulatory role in STING signaling activity by promoting STING degradation. As for the regulatory role of apoptosis in STING signaling activity including IFN and autophagy, it will be investigated in our future study.

## Data availability statement

The raw data supporting the conclusions of this article will be made available by the authors, without undue reservation.

## Author contributions

JZZ and WZ conceived and designed the experiments; NX, WZ, SJ, QC, JL, JJZ, YX, SS, KZ performed the experiments; WZ, NC, FM, JZZ analyzed the data; NX, WZ and JZZ wrote the paper. All authors contributed to the article and approved the submitted version.

## Funding

The work was partly supported by the National Natural Science Foundation of China (32202818; 32172867; 31872450), and A Project Funded by the Priority Academic Program Development of Jiangsu Higher Education Institutions (PAPD).

## Conflict of interest

The authors declare that the research was conducted in the absence of any commercial or financial relationships that could be construed as a potential conflict of interest.

The reviewer QL declared a shared affiliation with the author FM to the handling editor at the time of review.

## Publisher’s note

All claims expressed in this article are solely those of the authors and do not necessarily represent those of their affiliated organizations, or those of the publisher, the editors and the reviewers. Any product that may be evaluated in this article, or claim that may be made by its manufacturer, is not guaranteed or endorsed by the publisher.

## Supplementary material

Supplementary Figure 1–8 The densitometry analysis of the protein blots in, **Figures 1A–D**, **2G–I**, **3A, B**, **3E, F**, **4B**, **5B**, **5D**, **6A-B**, **7A, B**, **7E** and **8B-D** were presented in **Figure S1**, **Figure S2**–**S8**, respectively. **p* < 0.05 and ***p* < 0.01.

Supplementary Figure 9 The genomic DNA sequencing results of porcine Atg5 and Atg16L1 knockout cell clones. The Atg5 **(A–D)** and Atg16L1 **(E–G)** genomic PCR products of 3D4/21 clones were cloned into T vectors and the multiple cloned sequences were aligned with the corresponding Atg genome templates for analysis of base insertion/deletion (ins/del) mutations. For each cell clones, two typical sequences were shown for alignment, with the base ins (+) and del (-) indicated on the right. The red boxed Atg KO cell clones were selected for subsequent experiments.
