# Supplementary material for: Porcine cGAS-STING signaling induced autophagy inhibits STING downstream IFN and apoptosis
Source: Front Immunol. 2022 Oct 13;13:1021384. doi: 10.3389/fimmu.2022.1021384 (PMC9608012; doi:10.3389/fimmu.2022.1021384)
Supplement: Supplementary file 10 [file Table_1.doc]

**Supplementary Table 1**. PCR Primers for pSTING mutation and cloning

| **Primers** | **Sequence (5’-3’)** |
| --- | --- |
| pSTING S365A -F  pSTING S365A -R  pSTING L373A -F  pSTING L373A -R  pSTING ΔCTT -F  pSTING ΔCTT -R | 5'-CCTGAGCTCCTCATCGCTGGCATGGAACAGCC-3'  5'-GGCTGTTCCATGCCAGCGATGAGGAGCTCAGG-3'  5'-TATCTGAGCGGGCTGGAAGAGGCTGTTCCATGCC-3'  5'-GGCATGGAACAGCCTCTTCCAGCCCGCTCAGATA-3'  5'-TCGGCAGGAGGAAAGGGATATCACCAGCTACC-3'  5'-GGTAGCTGGTGATATCCCTTTCCTCCTGCCGA-3' |
| pSTING-pmCherry -F  pSTING-pmCherry -R | 5'-ACAAGTCCGGACTCAGATCTCCCTACTCCAGCCTGCATCCAT-3'  5'-GTACCGTCGACTGCAGAATTCTCAGAAGATATCTGAGCGGAG-3' |
| pSTING LIR1 mut-pcDNA DEST^47^ -F  pSTING LIR1 mut-pcDNA DEST^47^ -R  pSTING LIR1 mut-pmCherry -F  pSTING LIR1 mut-pmCherry -R  pSTING LIR2 mut -F  pSTING LIR2 mut -R  pSTING LIR3 mut -F  pSTING LIR3 mut -R  pSTING LIR4 mut -F  pSTING LIR4 mut -R  pSTING LIR5 mut -F  pSTING LIR5 mut -R | 5'-TCAGTCGACATGCCCGCCTCCAGCGCGCATCCATCCATCCC-3'  5'-GGGATGGATGGATGCGCGCTGGAGGCGGGCATGTCGACTGA-3'  5'-TGGGATGGATGGATGCGCGCTGGAGGCGGGCATCTTGTACAGC-3'  5'-GCTGTACAAGATGCCCGCCTCCAGCGCGCATCCATCCATCCCA-3'  5'-GGAGGCCAGGTGGGCCACCAGCGCCCGGAGCGTGTAT-3'  5'-ATACACGCTCCGGGCGCTGGTGGCCCACCTGGCCTCC-3'  5'-GCCAGCCTTGTCTCGGGCGGAGAAGGCGAAGTAGCAGGACAGC-3'  5'-GCTGTCCTGCTACTTCGCCTTCTCCGCCCGAGACAAGGCTGGC-3'  5'-GAGCCCTGGGAGGATCGCCCGCAGGGCCCCGATGTAATAAGAC-3'  5'-GTCTTATTACATCGGGGCCCTGCGGGCGATCCTCCCAGGGCTC-3'  5'-CGGCTGCCCGTTCTCCGCAAGCTCAGCGATGCTGTTGGTGTAC-3'  5'-GTACACCAACAGCATCGCTGAGCTTGCGGAGAACGGGCAGCCG-3' |

**Supplementary Table 2. CRISPR gRNA and Detection Primers for Homozygous KO 3D4/21 Cell Clones**

| **Primers** | **Sequence (5’-3’)** |
| --- | --- |
| pATG5 gRNA1 -F  pATG5 gRNA1 -R  pATG5 gRNA2 -F  pATG5 gRNA2 -R  pATG16L1 gRNA1 -F  pATG16L1 gRNA1 -R  pATG16L1 gRNA2 -F  pATG16L1 gRNA2 -R  pATG5 detection primer -F  pATG5 detection primer -R  pATG16L1 detection primer -F  pATG16L1 detection primer -R | 5'-CACCGAAGATGTGCTTCGAGATGTG-3'  5'-AAACCACATCTCGAAGCACATCTTC-3'  5'-CACCGGATGAGATAACTGAACGGG-3'  5'-AAACCCCGTTCAGTTATCTCATCC-3'  5'-CACCGACCGAGGGAAGACCGCGGCG-3'  5'-AAACCGCCGCGGTCTTCCCTCGGTC-3'  5'-CACCGTGGAAGCGCCACATCTCGG-3'  5'-AAACCCGAGATGTGGCGCTTCCAC-3'  5'-GGATCTGACTGACACTCTTC-3'  5'-GCTGATCTGTGTCTTCTAAT-3'  5'-AGCTGAGTGCTGGGGCTGCA-3'  5'-TCGGCCTCCGGTTCCCACTA-3' |

**Supplementary Table 3.** Primers used for qPCR in this study

| **Primers** | **Sequences (5’-3’)** |
| --- | --- |
| Porcine IFNβ-F | 5’-TGAGCATTCTGCAGTACCTGA-3’ |
| Porcine IFNβ-R | 5’-CCGGAGGTAATCTGTAAGTCTGT-3’ |
| Porcine ISG15-F | 5’-ATCCTGGTGAGGAACGACAA-3’ |
| Porcine ISG15-R | 5’-GAAAGTCAGCCAGAACTGGTC-3’ |
| Porcine CXCL10-F | 5’-CCCACATGTTGAGATCATTGC-3’ |
| Porcine CXCL10-R | 5’-CATCCTTATCAGTAGTGCCG-3’ |
| Porcine TNFα-F | 5’- ATCGCCGTCTCCTACCAGA-3’ |
| Porcine TNFα-R | 5’- TCGATCATCCTTCTCCAGCT-3’ |
| Porcine β-actin-F | 5’-ATGAAGATCAAGATCATCGCG-3’ |
| Porcine β-actin-R | 5’-TCGTACTCCTGCTTGCTGATC-3’ |
